# Health System Integration and Equity in Type 2 Diabetes Prevention: A Comparative Policy Analysis of Seven National Guidelines

**DOI:** 10.1111/jep.70591

**Published:** 2026-09-08

**Authors:** Richard Marfo, Dennis Miezah, Rockson Ansong, Musah Abubakari, Evans F. Kyei

**Affiliations:** ^1^ Department of Nursing University of Massachusetts Boston Boston Massachusetts USA; ^2^ Louisiana State University Baton Rouge Louisiana USA; ^3^ Capstone College of Nursing, University of Alabama Tuscaloosa Alabama USA

**Keywords:** comparative policy analysis, health equity, health policy, health system integration, prevention, type 2 diabetes mellitus

## Abstract

**Rationale:**

Type 2 diabetes mellitus affects more than 500 million adults worldwide, yet up to 80% of related complications are preventable through evidence‐based care. Clinical practice guidelines function as key prevention policy instruments; however, their implementation and integration into health systems vary substantially, with important implications for prevention access and health equity.

**Aims and Objectives:**

This study aimed to examine how national policy integration architecture within diabetes management guidelines influences access to preventive care and health equity across diverse health system contexts.

**Methods:**

A comparative qualitative policy analysis evaluated national diabetes management guidelines from seven countries (United States, United Kingdom, Japan, India, Ghana, Colombia, Australia) using the Health Policy Triangle and Integration Continuum Framework. Guidelines were scored on integration strength (1 to 5 scale) across clinical, financial and administrative domains. Equity analysis examined financing barriers, workforce distribution and cultural adaptations. Inter‐rater reliability testing achieved substantial agreement (Cohen's kappa 0.68).

**Results:**

All guidelines converged on evidence‐based clinical content, yet integration scores varied threefold (range 2 to 5). High‐integration systems (United Kingdom, Australia; scores 5) achieved prevention medication access rates exceeding 80% among eligible populations through unified financing and zero cost‐sharing. Partial‐integration systems (United States, India, Ghana; scores 2 to 3) showed 30% to 50% access despite comparable clinical recommendations. Integration strength correlated strongly with implementation effectiveness (Spearman *ρ* = 0.89, *p* < 0.01). Financing structure emerged as the primary equity determinant. Countries with fragmented payment systems preserved disparities despite equity language in guidelines.

**Conclusions:**

Preventing diabetes complications at scale requires integrated health systems where financing, workforce, and monitoring align with guideline intent. Policy priorities should emphasise unified financing, elimination of cost‐sharing for preventive therapies, and mandatory equity monitoring in quality frameworks.

## 1 Introduction

Type 2 diabetes mellitus (T2DM) affects more than 500 million adults globally, yet up to 80% of complications are preventable through evidence‐based care [1]. Clinical practice guidelines serve as prevention policy instruments, translating evidence into recommendations for screening, treatment and monitoring. However, clinical evidence alone does not ensure population benefit. Guideline implementation depends on financing structures, workforce capacity and monitoring systems that vary dramatically across health systems [2]. This comparative analysis examines how seven national diabetes guidelines translate evidence into practice through different integration models, with particular attention to prevention access and health equity. Global consensus on diabetes management has accelerated since 2010. Clinical guidelines now universally prioritise cardio‐renal protective therapies such as SGLT2 inhibitors and GLP‐1 receptor agonists for high‐risk individuals [3]. Most guidelines recommend metformin as first‐line therapy, individualised HbA1c targets and structured lifestyle interventions. Yet adoption remains uneven. Cost barriers, infrastructure gaps and workforce constraints limit diffusion of life‐saving preventive medications, particularly in low‐ and middle‐income countries [4]. This paradox between clinical consensus and implementation divergence suggests that guideline content alone cannot explain prevention effectiveness. System integration may matter more than evidence quality.

The Health Policy Triangle (HPT) provides a structured framework for understanding this implementation gap [5]. The HPT analyses policy through four interrelated dimensions: actors (stakeholders and institutions), context (political, economic and social environment), content (technical guidance and recommendations) and process (development, dissemination and evaluation mechanisms). The framework emphasises that health policy is not purely technical but embedded in governance structures and institutional capacity [6]. Applying the HPT to national diabetes guidelines reveals how decisions about financing, workforce deployment and quality monitoring reflect broader health system characteristics. Recent methodological advances recommend combining the HPT with additional frameworks to address its limitations in explaining implementation outcomes [7]. The Integration Continuum Framework complements the HPT by assessing coordination across clinical, financial and administrative domains [8]. The continuum rates systems from fragmented (score 1), where actors operate independently, to fully integrated (score 5), where clinical protocols, payment mechanisms and monitoring function cohesively. Integration directly affects prevention outcomes. Fragmented financing creates cost barriers to preventive medications and screening. Workforce gaps delay diagnosis and treatment intensification. Disconnected monitoring systems miss opportunities to identify high‐risk individuals or intervene before complications develop. The dual‐framework approach positions guidelines as system interventions rather than technical documents, examining how policy architecture enables or blocks prevention at scale.

This comparative analysis examines seven national diabetes guidelines to determine how integration architecture shapes prevention access and equity. Existing comparative studies focus on cost‐effectiveness or clinical outcomes without examining integration mechanisms [9]. This study fills that gap. For prevention practice, this analysis identifies which system features enable population‐wide implementation of complication‐preventing therapies. For policy makers, it demonstrates how integration architecture predicts equitable access to preventive services. The findings inform prevention strategies in both high‐income settings struggling with fragmentation and low‐ and middle‐income countries building chronic disease programmes. As diabetes prevalence continues rising globally, understanding how to translate evidence into equitable prevention becomes increasingly urgent.

## 2 Methods

### 2.1 Study Design and Frameworks

This study employed a comparative qualitative policy analysis design to examine national type 2 diabetes management guidelines across seven countries: United States, United Kingdom, Japan, India, Ghana, Colombia and Australia. These countries were selected to represent diverse income levels, governance arrangements and equity challenges. Selection criteria prioritised geographic diversity, income variation based on World Bank classifications, and distinct approaches to equity. Australia addresses Indigenous health through dedicated pathways, India confronts caste and regional disparities, Ghana manages rural access constraints and the United States and Colombia face insurance‐based inequities despite universal coverage frameworks. This variation allows examination of how different integration models affect prevention access across population groups. The design enabled systematic comparison of guideline structures, implementation mechanisms and integration within diverse health system contexts. This analysis aligns with Preventive Medicine's equity framework by examining how integration patterns affect access to preventive services across population groups, with particular attention to financing barriers, geographic distribution and culturally adapted care. Two complementary frameworks guided data collection and analysis. The Health Policy Triangle (HPT) framework [5] structured extraction across four domains: actors (institutions and stakeholders responsible for policy formulation), context (political, economic and social environments shaping policy), content (technical and operational recommendations) and process (guideline development, dissemination and evaluation mechanisms). The HPT provided a structural lens for understanding how policy decisions emerge, and which system features influence implementation [6]. The Integration Continuum Framework [8] assessed coordination across clinical, financial and administrative domains. Each guideline received an integration score from 1 (minimal/fragmented integration) to 5 (comprehensive integration across system components). This framework complemented the HPT by revealing how policy design translates into operational practice, particularly for prevention delivery.

### 2.2 Data Sources

Primary documents included the most recent authoritative national diabetes management guidelines for each country: ADA Standards of Care in Diabetes [10] (United States), NICE NG17 [11] update (United Kingdom), Japan Diabetes Society [12] Guidelines, ICMR [13] Guidelines (India), Ghana Ministry of Health [14] Guidelines, Colombia Ministry of Health [15] Guideline and RACGP Diabetes Handbook [16] (Australia). All documents were obtained from official websites of issuing agencies or national health authorities. Supporting documents reviewed for triangulation included QOF audit reports (United Kingdom, *n* = 3), NPCDCS state implementation reviews (India, *n* = 5), EPS performance audits (Colombia, *n* = 4), RACGP chronic disease item reports (Australia, *n* = 2) and HEDIS measure specifications (United States, *n* = 6). Document dates ranged from 2020 to 2024 to ensure currency. Data were cross‐checked against WHO country profiles and published health system reviews to contextualise implementation structures, workforce distribution and financing models [17].

### 2.3 Data Extraction and Coding

A structured data extraction matrix organised information by framework domain. For the HPT, data were coded into actors, context, content and process categories. For the Integration Continuum, indicators were categorised under clinical integration (multidisciplinary care models, continuity mechanisms, quality monitoring), financial integration (reimbursement alignment, coverage scope, cost‐sharing) and administrative integration (data systems, registries, audit mechanisms). For equity analysis, coders extracted: (a) explicit equity language in guidelines, (b) financing mechanisms for underserved populations, (c) rural and remote service provisions, (d) cultural adaptations, (e) task‐shifting strategies and (f) technology access provisions. Each guideline was read in full and summarised under these categories. A second pass ensured consistency of interpretation and identification of implicit features not explicitly stated in documents.

### 2.4 Scoring Criteria

Integration Continuum scores were assigned based on explicit criteria developed from Heath et al. [8] and adapted for diabetes policy contexts:

Score 1 (Minimal Integration): No multidisciplinary care standards in clinical domain. Fragmented coverage with high out‐of‐pocket costs (greater than 25% of total care costs) in financial domain. Manual or absent data systems in administrative domain.

Score 2 (Partial Integration): Guidelines mention multidisciplinary teams but provide no funding mechanisms. Universal coverage exists with major gaps such as newer cardio‐renal protective agents excluded. Basic registries with irregular audits (annual or less frequent).

Score 3 (Functional Integration): Funded multidisciplinary models operate in some regions but not standardised nationally. Comprehensive coverage with moderate cost‐sharing (10% to 25% of costs). Electronic registries with annual reporting to providers.

Score 4 (Advanced Integration): Standardised multidisciplinary care with structured training programmes. Unified coverage with minimal cost‐sharing (less than 10% of costs). Real‐time dashboards with feedback mechanisms to providers and facilities.

Score 5 (Full Integration): Seamless coordination across all care levels with role clarity. Zero cost‐sharing for preventive services and essential medications. Linked data systems enabling outcome‐based payment and population health management.

Implementation strength scores (1 to 5) captured the degree to which guidelines were institutionalised and operationalized across health system levels, based on documented workforce strategies, financing mechanisms and monitoring systems.

### 2.5 Inter‐Rater Reliability

To ensure scoring reliability, two researchers independently rated each country using the criteria above. Initial agreement was 71% across 42 indicators (6 indicators per country, 7 countries). Discrepancies were resolved through discussion and review of source documents. Final Cohen's kappa was 0.68, indicating substantial agreement. The primary researcher remained unaware of implementation outcome data during initial scoring to reduce confirmation bias.

### 2.6 Analytical Process

Cross‐case analytical synthesis identified patterns and contrasts across countries. Guided by the HPT, each country's policy was analysed for how actors, context, content and process interacted to facilitate or constrain integration. Findings were organised into thematic clusters: governance structures, financing alignment, workforce distribution, technology integration, and equity orientation. Integration scores were cross‐tabulated with equity indicators to assess whether high integration correlated with equitable implementation. Comparative matrices were developed to display differences in policy structure, integration levels, and implementation strength. Analytical memos linked descriptive findings with theoretical constructs from both frameworks.

### 2.7 Methodological Rigour

Rigour was ensured through: (1) dual‐framework application with explicit linkages, (2) triangulation across guideline documents and implementation reports, (3) transparent scoring with detailed criteria and (4) iterative coding with discrepancy resolution. Researcher positionality: the analysis team includes health policy researchers with expertise in health systems, comparative policy analysis, and diabetes care delivery. This diversity informed interpretation of context‐specific implementation features. Sex and gender considerations: This analysis adhered to SAGER guidelines [18]. While diabetes prevalence varies by sex, guideline content did not systematically address sex‐specific prevention or treatment needs. We noted which guidelines included pregnancy‐specific protocols or acknowledged gender differences in care‐seeking behaviours. Implementation data were not stratified by sex, representing a limitation for equity analysis. Guideline selection avoided disproportionate focus on high‐income countries to prevent reproducing global health knowledge inequities.

## 3 Results

### 3.1 Overview of Integration and Implementation

Seven national diabetes guidelines demonstrated striking clinical convergence. All recommend metformin as first‐line therapy, individualised HbA1c targets near 7%, and cardio‐renal protective agents (SGLT2 inhibitors, GLP‐1 receptor agonists) for high‐risk individuals. Yet integration strength diverged dramatically. Integration Continuum scores ranged from 2 (India) to 5 (United Kingdom, Australia), with implementation strength closely correlated (Spearman *ρ* = 0.89, *p* < 0.01). High‐integration systems eliminated cost barriers to preventive medications and achieved equitable primary care access, while fragmented systems preserved disparities despite clinical consensus (Table 1). Countries clustered into three integration patterns. High‐integration systems (UK, Australia; scores 5) combined unified financing with zero cost‐sharing for diabetes care. Advanced‐integration systems (Japan, score 4; Colombia, score 3) achieved functional coordination through standardised fee schedules or audit‐linked reimbursement. Partial‐integration systems (United States, Ghana, India; scores 2–3) faced structural fragmentation in financing, workforce distribution, or monitoring capacity despite sophisticated clinical guidance.

**Table 1:** Integration and implementation characteristics of national type 2 diabetes guidelines across seven countries.

Country	Health system type	Integration score (1–5)	Implementation strength (1–5)	Primary integration driver	Primary fragmentation barrier
United Kingdom	Single payer (NHS)	5	5	QOF performance metrics linked to GP payment; unified NHS financing eliminates cost barriers	Regional workforce distribution gaps; specialist wait times in some areas
Australia	Single payer (Medicare)	5	5	PBS drug subsidies provide zero cost‐sharing; MBS chronic disease item numbers fund team care	Rural and remote access constraints; cross‐jurisdiction data linkage incomplete
Japan	Single payer (NHI)	4	4	Uniform national fee schedule standardises care delivery; certified diabetes educator network	Rapid population aging; rural specialist shortage; limited weight management services
Colombia	Regulated insurers (EPS)	3	4	EPS audit‐linked reimbursement ties payment to guideline adherence; national quality indicators	Persistent inequities between contributory and subsidised insurance regimes
United States	Multi‐payer private/public	3	4	Advanced technology access (CGM, insulin pumps); multiple evidence‐based guidelines	Fragmented payer coverage; high patient cost‐sharing ($300‐900/month for newer agents); variable reimbursement for team care
Ghana	Mixed public/NHIS	3	3	Pragmatic level‐of‐care algorithm enables task‐shifting; explicit fasting and pregnancy protocols	NHIS excludes cardio‐renal protective agents; supply chain interruptions; limited laboratory capacity
India	Mixed public‐private	2	2	Lower BMI screening threshold (≥ 23 kg/m^2^) addresses ethnic risk; task‐shifting to nurses and CHWs	Ayushman Bharat limited outpatient drug coverage; heterogeneous private sector quality; weak real‐time monitoring

*Note:* Integration scores reflect coordination across clinical, financial and administrative domains based on explicit criteria (see Methods). Implementation strength indicates degree of institutionalization across health system levels.

Abbreviations: CHW, community health worker; CGM, continuous glucose monitoring; EPS, Entidades Promotoras de Salud; GP, general practitioner; MBS, Medicare Benefits Schedule; NHI, National Health Insurance; NHIS, National Health Insurance Scheme; NHS, National Health Service; PBS, Pharmaceutical Benefits Scheme; QOF, Quality and Outcomes Framework.

### 3.2 High‐Integration Systems: United Kingdom and Australia

The United Kingdom and Australia achieved comprehensive integration through single‐payer financing models that aligned clinical protocols with payment mechanisms and quality monitoring [19]. The UK's Quality and Outcomes Framework directly linked guideline adherence to general practitioner payment, incentivizing annual diabetes reviews including HbA1c measurement, blood pressure monitoring and foot examinations. NHS prescription charge exemptions for diabetes ensured zero cost‐sharing for all medications, enabling rapid adoption of cardio‐renal protective therapies. More than 80% of eligible patients received SGLT2 inhibitors or GLP‐1 receptor agonists within 24 months of guideline updates. Multidisciplinary teams operated as standard practice, with practice nurses, pharmacists and diabetes specialist nurses integrated into primary care delivery. Australia's Medicare Benefits Schedule and Pharmaceutical Benefits Scheme created similar integration architecture [20]. PBS subsidies capped patient costs at AU$31.60 per prescription with annual safety nets at AU$277.20, functionally eliminating cost barriers for chronic disease management. MBS chronic disease management item numbers reimbursed extended consultations and care planning, funding team‐based approaches. The RACGP guideline explicitly addressed Aboriginal and Torres Strait Islander health through dedicated pathways and funded Aboriginal Health Worker programmes, demonstrating equity‐oriented integration design. Both systems maintained real‐time electronic registries enabling provider‐level performance feedback. However, equity gaps persisted. The UK faced regional workforce shortages and specialist wait times in underserved areas. Australia's prevention access rates among Indigenous populations (65%) lagged behind non‐Indigenous populations (> 80%), despite dedicated programmes, reflecting ongoing implementation challenges in remote communities [21].

### 3.3 Japan: Functional Integration Through Uniform Standards

Japan achieved advanced integration (score 4) through a different mechanism: uniform national insurance fee schedules standardising care delivery nationwide. All providers received identical reimbursement for diabetes services regardless of geography or facility type, ensuring pricing equity. The Japan Diabetes Society guideline embedded certified diabetes educator roles within hospital‐community networks, extending specialist expertise to primary care settings. National Database of Health Insurance Claims enabled population‐level tracking and society‐led audits. Implementation strength was high (score 4), with consistent guideline adoption across prefectures. However, rapid population aging and rural specialist shortages constrained service delivery in remote areas. The guideline emphasised postprandial glucose control, aligning with traditional dietary patterns, but limited integration of weight management services reflected structural gaps in obesity pharmacotherapy access.

### 3.4 Partial‐Integration Systems: Diverse Fragmentation Patterns

The United States demonstrated an integration paradox: technologically advanced clinical guidance within structurally fragmented financing. The ADA Standards of Care 2024 provided comprehensive, annually updated recommendations with rigorous evidence grading. Multiple payers adopted guideline‐concordant coverage, enabling high implementation strength (score 4) for insured populations. However, fragmented coverage created marked inequities. Patient cost‐sharing ranged from $300 to $900 monthly for GLP‐1 receptor agonists despite clinical indication. Prior authorization requirements delayed therapy initiation. Prevention access rates of 40% to 50% among eligible populations reflected financial barriers rather than guideline quality. Racial disparities were pronounced: White adults achieved 35% continuous glucose monitoring access compared to 18% among Black adults, despite equivalent clinical indications. Colombia achieved moderate integration (score 3) through its Entidades Promotoras de Salud model. Audit‐linked reimbursement tied insurer payments to guideline adherence and quality indicators, creating administrative integration. However, persistent inequities between contributory and subsidised insurance regimes limited effectiveness. Patients in contributory regimes accessed cardio‐renal protective therapies at 2.3 times the rate of subsidised regime enrollees, demonstrating how administrative integration without financial equity preservation perpetuates disparities.

Ghana and India illustrated challenges facing low‐ and middle‐income countries [22]. Both guidelines demonstrated pragmatic adaptation to resource constraints. Ghana's level‐of‐care algorithm explicitly addressed task‐shifting, enabling nurses and medical officers to manage uncomplicated diabetes at primary care level [23]. Ramadan fasting protocols and pregnancy‐specific guidance reflected cultural responsiveness. However, National Health Insurance Scheme exclusion of SGLT2 inhibitors and GLP‐1 receptor agonists limited cardio‐renal protection to 30% to 40% of indicated populations. Supply chain interruptions and limited laboratory capacity further constrained implementation. India's ICMR guideline incorporated lower BMI thresholds (≥ 23 kg/m^2^) acknowledging ethnic risk patterns and integrated AYUSH practices reflecting cultural context. Task‐shifting to community health workers extended screening reach [24]. Yet Ayushman Bharat coverage excluded outpatient medications, requiring out‐of‐pocket payment for essential drugs. Heterogeneous private sector quality and weak real‐time monitoring systems resulted in implementation scores of 2, the lowest among studied countries. Wide state‐level variation reflected India's federal structure, with some states achieving functional integration through dedicated programmes while others maintained minimal coordination.

### 3.5 Cross‐Cutting Patterns

Three patterns emerged across integration levels. First, financing structure determined prevention access more than guideline content quality. Single‐payer systems with unified formularies (UK, Australia, Japan) achieved 70% to 80% prevention medication access. Multi‐payer or resource‐limited systems showed 30% to 50% access despite comparable clinical recommendations. Second, workforce integration enabled sustained implementation. Countries institutionalising multidisciplinary care with funded roles (UK practice nurses, Australia chronic disease item numbers, Japan certified educators) maintained higher screening rates (> 75% annually) compared to opportunistic screening models (30% to 50%). Third, real‐time monitoring systems distinguished high‐performing from partial‐integration systems. Electronic registries with provider feedback (UK, Australia, Japan) enabled continuous quality improvement, while manual or absent systems (India, Ghana) prevented identification of care gaps.

### 3.6 Equity Analysis: Integration as Prerequisite for Equitable Prevention

Equity provisions and prevention access varied systematically by integration level (Table 2). High‐integration systems eliminated financial barriers, achieving prevention access rates above 80% among eligible populations. Australia's explicit Aboriginal and Torres Strait Islander pathways demonstrated equity‐oriented design, though implementation gaps persisted in remote areas. Japan's uniform fee schedule ensured geographic equity in core services. Partial‐integration systems showed persistent inequities despite equity language in guidelines. The United States included social determinants assessment in ADA Standards, but fragmented coverage preserved race‐based and income‐based access disparities. Colombia's universal EPS model existed on paper, yet contributory‐subsidised inequities limited cardio‐renal protective therapy access. Ghana and India adapted guidelines culturally (fasting protocols, lower BMI thresholds, AYUSH integration) but lacked financing mechanisms to operationalise equity intent. Cost barriers to preventive therapies emerged as the dominant equity determinant. Countries with comprehensive formulary coverage and minimal cost‐sharing achieved treatment penetration exceeding 80% among eligible populations [25]. Those with significant out‐of‐pocket requirements showed 30% to 50% penetration despite clinical guidelines recommending universal access for high‐risk individuals. Integration architecture thus functioned as infrastructure enabling or blocking equitable prevention, independent of guideline technical quality.

**Table 2:** Equity‐oriented policy provisions and prevention access patterns in national diabetes guidelines.

Country	Explicit equity provisions	Cost barriers to cardio‐renal protective therapies	Rural/remote access mechanisms	Cultural adaptation features	Prevention access rate (eligible population)^a^
United Kingdom	Universal NHS access; no explicit ethnicity‐based provisions	None (zero cost‐sharing for all diabetes medications via NHS prescription charges exemption)	Community diabetes hubs in underserved areas; telehealth for remote consultations	Pregnancy and Ramadan fasting guidance	> 80% (SGLT2i/GLP‐1 RA among indicated patients)
Australia	Dedicated Aboriginal and Torres Strait Islander health pathways; funded Aboriginal Health Worker programmes	None (PBS subsidies cap patient costs at $31.60/prescription; safety net at $277.20/year)	Mobile screening services; extended telehealth reimbursement for rural areas	Aboriginal Health Worker integration; culturally adapted primary care protocols	> 80% (Indigenous patients 65%; ongoing gap‐closing initiatives)
Japan	Uniform fee schedule ensures geographic equity in pricing	Minimal (national insurance covers 70%–90% of costs; monthly out‐of‐pocket caps)	Hospital‐community networks extend specialist access; regional diabetes centres	Postprandial glucose emphasis aligns with dietary patterns	70%–75% (rural access lower due to specialist distribution)
Colombia	EPS model provides universal coverage on paper	Moderate (newer agents available but formulary variation across EPS; contributory regime 2.3× access rate vs. subsidised)	Referral protocols link primary care to regional centres	Guidelines available in Spanish; emphasis on tropical disease comorbidities	50%–60% (marked contributory/subsidised disparity)
United States	ADA Standards include social determinants section; emphasises screening for food/housing insecurity	High (fragmented coverage; typical patient copays $300–900/month for GLP‐1 RA; prior authorization requirements)	Telehealth expansion post‐COVID; rural health clinic incentives	Guidelines acknowledge race‐based disparities; no mandatory culturally adapted protocols	40%–50% (White adults 35% CGM access; Black adults 18%; income‐based disparities)
Ghana	Task‐sharing algorithm designed for resource‐limited settings; explicit Ramadan fasting protocols	Very High (NHIS excludes SGLT2i and GLP‐1 RA entirely; patients pay full cost out‐of‐pocket)	Level‐of‐care tiered approach enables primary care management	Fasting guidance for Muslim populations; pregnancy protocols adapted to local context	30%–40% (limited to metformin, sulfonylureas, insulin)
India	Lower BMI threshold (≥ 23 kg/m^2^) for screening; integration of AYUSH practices	Very High (Ayushman Bharat covers inpatient only; outpatient drugs require out‐of‐pocket payment)	NPCDCS state‐level programmes variable; CHW home visits in some states	Yoga and lifestyle modifications culturally grounded; regional language adaptations	30%–40% (wide state‐level variation; private sector access for wealthy)

Abbreviations: AYUSH, Ayurveda, Yoga, Unani, Siddha, Homeopathy; CHW, community health worker; CGM, continuous glucose monitoring; EPS, Entidades Promotoras de Salud; GLP‐1 RA, glucagon‐like peptide‐1 receptor agonist; NHIS, National Health Insurance Scheme; NHS, National Health Service; NPCDCS, National Program for Prevention and Control of Cancer, Diabetes, Cardiovascular Diseases and Stroke; PBS, Pharmaceutical Benefits Scheme; SGLT2i, sodium‐glucose cotransporter 2 inhibitor.

^a^ Prevention access rates represent estimated percentage of clinically eligible populations receiving cardio‐renal protective therapies (SGLT2 inhibitors or GLP‐1 receptor agonists) based on guideline documents, audit reports and published implementation studies where available. Rates are approximations derived from heterogeneous data sources.

## 4 Discussion

### 4.1 Principal Findings

Three key findings emerge from this seven‐country comparison. First, clinical consensus has not produced implementation convergence. Despite identical evidence‐based content, integration scores varied threefold (range 2 to 5). Second, financing structure, specifically unified versus fragmented payment systems, emerged as the primary determinant of both integration strength and equity outcomes. Third, guideline content emphasised prevention through HbA1c control and cardio‐renal protection, yet systems varied dramatically in translating prevention intent into population access. These findings advance comparative diabetes policy research by positioning guidelines as system interventions rather than technical documents [9]. Prior cross‐national studies focused on clinical outcomes or cost‐effectiveness without examining integration mechanisms [26]. This analysis demonstrates that policy architecture shapes prevention effectiveness as strongly as evidence quality. The dual‐framework approach revealed a paradox: actor coordination and financing integration predict outcomes more powerfully than clinical guideline sophistication. High‐integration systems achieved prevention medication access rates exceeding 80% among eligible populations, while fragmented systems reached only 30% to 50% despite comparable clinical recommendations. This gap represents millions of preventable cardiovascular events and kidney failure cases globally. These findings both align with and extend prior comparative policy research. Allen et al. [26] analysed non‐communicable disease policy implementation across 151 countries and similarly found that policy adoption did not translate uniformly into implementation strength, a pattern consistent with the threefold variation in integration scores observed here despite convergent clinical content. Seghieri et al. [9] compared diabetes and hypertension policies across European countries and reported that financing and governance structures, rather than guideline content, explained most cross‐country variation in care quality; the present findings extend this observation beyond Europe to a broader set of income contexts, showing that the same financing‐driven pattern holds across single‐payer, multi‐payer and mixed public‐private systems. Similarly, Owolabi et al. [4] identified systematic gaps between low‐ and middle‐income and high‐income country guidelines, attributing these primarily to resource constraints rather than clinical knowledge gaps, which corresponds with this study's finding that Ghana and India achieved lower integration scores despite guidelines that were culturally adapted and clinically sound. Where this analysis diverges from prior work is in its explicit quantification of integration strength using a structured scoring framework linked to implementation and equity outcomes; earlier comparative studies, including Brusamento et al. [2] and Martínez‐González et al. [19], examined implementation strategies or integrated care models in general terms without a comparable scoring mechanism connecting policy architecture directly to prevention access rates. This methodological distinction may explain why the present study identifies financing structure as a more powerful predictor of equity outcomes than prior reviews, which tended to treat financing as one implementation barrier among several rather than as the primary determinant.

### 4.2 Integration as Prevention Infrastructure

Integration functions as prevention infrastructure through three mechanisms. First, financial integration eliminates cost barriers to preventive therapies. High‐integration systems provided zero or minimal cost‐sharing for SGLT2 inhibitors and GLP‐1 receptor agonists in patients with cardiovascular or renal risk. These agents prevent heart failure, kidney failure and mortality [27]. The UK's NHS prescription exemptions and Australia's PBS subsidies achieved treatment penetration above 80% among indicated populations. In contrast, US patients faced monthly copays of $300 to $900, India's Ayushman Bharat excluded outpatient drugs, and Ghana's NHIS did not cover newer agents. These financial barriers limited prevention access to 30% to 50% of indicated populations despite clinical guidelines recommending universal use.

Second, workforce integration enables early detection and treatment intensification. High‐integration systems institutionalised multidisciplinary teams with funded time for screening, education and medication titration [28]. The UK's QOF incentivized comprehensive annual diabetes reviews. Australia's MBS funded extended chronic disease consultations. These mechanisms achieved annual screening rates above 75%. Partial‐integration systems relied on opportunistic screening with lower capture rates (40% to 50% in India and Ghana), delaying diagnosis and complication prevention. Task‐shifting extended reach in resource‐limited settings but remained under‐resourced without corresponding financing integration [24].

Third, monitoring integration enables continuous quality improvement. High‐integration systems used electronic registries with provider‐level feedback loops [29]. The UK's NHS dashboards identified underperforming practices for targeted support. Japan's National Database enabled population‐level tracking. Partial‐integration systems lacked real‐time data, preventing identification of prevention gaps. These integration mechanisms directly affect preventable outcomes. Modelling studies estimate that guideline‐concordant SGLT2 inhibitor and GLP‐1 receptor agonist use could prevent 20% to 30% of cardiovascular events and 40% of kidney failure progression in T2DM populations. This analysis suggests less than 50% of prevention potential is realised in partial‐integration systems.

### 4.3 Equity as Integration Outcome

Equity analysis showed that high integration is necessary but insufficient for equitable prevention. Australia and the UK achieved strongest equity through explicit mechanisms: Aboriginal Health Worker programmes, zero cost sharing and equity monitoring in quality frameworks [25]. However, residual disparities persisted in rural areas and among Indigenous populations, demonstrating that even highly integrated systems require ongoing equity‐focused interventions. Partial‐integration systems showed persistent inequities despite equity language in guidelines. The United States included social determinants assessment in clinical guidance, but fragmented coverage preserved race‐based and income‐based disparities [30]. This disconnect between policy intent and operational reality characterises many health systems transitioning from volume‐based to value‐based care without corresponding financing reform.

### 4.4 Policy Implications

For high‐income settings, prevention effectiveness requires addressing residual fragmentation. The United States should cap patient cost‐sharing for diabetes medications, reimburse team‐based diabetes self‐management education through Medicare and Medicaid, and mandate interoperable registries across payers. Rural access gaps in the UK, Australia and Japan require expanded telehealth reimbursement and incentives for rural practitioner distribution. For low‐ and middle‐income countries, integration can be achieved through pragmatic adaptations. Pooled procurement for essential diabetes medications at national or regional levels reduces costs [31]. Eliminating user fees for diabetes care and monitoring removes financial barriers. Negotiating tiered pricing for cardio‐renal protective agents through WHO prequalification mechanisms expands access. Formalising task‐shifting with reimbursement for nurse‐led medication titration, pharmacy therapy management and community health worker adherence support extends workforce capacity [32]. For all settings, equity‐oriented integration requires zero cost‐sharing for preventive medications and monitoring, mandatory equity stratification in quality metrics by geography and sociodemographic, culturally adapted protocols developed with community input, and workforce representation from underserved populations.

### 4.5 Limitations

This analysis has three main limitations. First, guideline analysis reflects policy intent rather than implementation reality. Guideline existence does not ensure provider adherence or patient access. Second, integration scores represent relative rankings rather than absolute thresholds. A score of 5 does not indicate perfect integration but rather the highest level observed among the seven countries studied. Third, cross‐sectional analysis cannot establish causation. Longitudinal studies tracking integration changes and outcome trends would strengthen causal inference. Additionally, some countries (India, Ghana, Colombia) provided limited publicly available data on financing and monitoring systems, requiring interpretation from supplementary sources.

## 5 Conclusion

Preventing diabetes complications at scale requires more than clinical evidence. It demands integrated health systems where financing, workforce and monitoring align with guideline intent. High‐integration systems eliminated cost barriers and achieved equitable access to life‐saving preventive therapies, while fragmented systems perpetuated disparities despite clinical consensus. As diabetes prevalence continues rising globally, the policy priority is clear: strengthen integration architecture through unified financing, workforce investment and data infrastructure. Evidence‐based guidelines are necessary, but systems integration is sufficient for prevention effectiveness.

## Funding

The authors have nothing to report.

## Ethics Statement

This study involved the analysis of publicly available policy documents that had received prior institutional ethical approval. As no human participants or identifiable data were involved, additional ethical approval was not required.

## Conflicts of Interest

The authors declare no conflicts of interest.

## Data Availability

All the data analysed or generated in this study are contained in this article.
